# Multiparametric Ultrasound Assessment of Long-Term Liver Damage in COVID-19: Results of a Three-Year Follow-Up Study

**DOI:** 10.3390/medicina62061077

**Published:** 2026-06-02

**Authors:** Maija Radzina, Davis Simanis Putrins, Ieva Vanaga, Oksana Kolesova, Arvids Buss, Aija Agera, Ludmila Viksna

**Affiliations:** 1 Institute of Diagnostic Radiology, Pauls Stradins Clinical University Hospital, LV-1002 Riga, Latvia; 2 Department of Radiology, Faculty of Medicine, Rīga Stradiņš University, LV-1007 Riga, Latvia; 3 Department of Infectology, Rīga Stradiņš University, LV-1007 Riga, Latvia; 4 Institute of Microbiology and Virology, Rīga Stradiņš University, LV-1007 Riga, Latvia; 5 Riga East Clinical University Hospital, Infectology Center, LV-1007 Riga, Latvia; 6 Department of Residency, Rīga Stradiņš University, LV-1007 Riga, Latvia; 7 Faculty of Medicine and Life Sciences, University of Latvia, LV-1004 Riga, Latvia

**Keywords:** multiparametric ultrasound, liver damage, COVID-19

## Abstract

***Background:*** Liver damage in COVID-19 is multifaceted, including liver steatosis and inflammation. Multiparametric ultrasound (mpUS) is an imaging modality that has the capacity to evaluate various facets of overall liver health and is relatively accessible and thus is an excellent choice to determine parenchymal changes. ***Methods:*** A longitudinal prospective study was designed to evaluate long-term hepatic damage following COVID-19 using mpUS. Patients were assessed within a 3–6-month period after the initial episode of COVID-19 and subsequently had a follow-up after 3 years compared to a control group. Liver stiffness (2D-SWE), attenuation (ATI), and shear wave dispersion (SWD) were measured to quantify liver stiffness, steatosis, and tissue viscosity. ***Results:*** A total of 129 patients were scanned at the baseline assessment, 90 patients in research group (58 patients had a follow-up) and 39 as a clinically healthy control group, and all were included in the follow-up for evaluation after 3 years. The mpUS evaluation in research group revealed a median SWE decrease from 5.04 ± 1.74 kPa to 4.59 ± 0.81 kPa and SWD decrease from 11.88 ± 1.73 m/s/kHz to 10.83 ± 1.49 m/s/kHz (*p* > 0.05); in contrast, median ATI values showed slight increase over time—0.56 ± 0.09 dB/cm/MHz to 0.60 ± 0.09 dB/cm/MHz (*p* > 0.05). Control group was stratified according to subsequent COVID-19 status. In both the COVID-negative and -positive subgroups SWE slightly increased from initial 4.55 ± 0.78 kPa to 4.8 ± 0.88 kPa and 4.7 ± 1.29 kPa, median SWD had a slight decrease from initial 10.80 ± 1.73 m/s/kHz to 10.15 ± 1.87 m/s/kHz and 10.6 ± 1.82 m/s/kHz (*p* > 0.05), and ATI increased significantly from initial 0.57 ± 0.08 dB/cm/MHz to 0.62 ± 0.09 dB/cm/MHz and 0.65 ± 0.07 dB/cm/MHz (*p* < 0.05), respectively. ***Conclusions:*** The study found that initially COVID-19-affected patients showed stable ATI and BMI values, no hepatic steatosis, normal SWE, and reduced dispersion which suggests resolving inflammation without fibrosis. Controls showed increased ATI and mild steatosis, likely linked to BMI and metabolic changes rather than direct viral liver injury.

## 1. Introduction

Long-term hepatic injury and fibrosis have emerged as significant post-COVID-19 sequelae, associated with metabolic, immune, and endothelial dysfunction triggered by SARS-CoV-2 infection [1,2,3,4,5]. Viral-induced activation of Kupffer cells and hepatic stellate cells may result in excessive extracellular matrix deposition and sustained fibrogenic activity [2,4]. Beyond direct viral effects, behavioral changes during the pandemic, including reduced physical activity, increased alcohol consumption, and higher calorie intake, have contributed to the global rise in metabolic dysfunction-associated steatotic liver disease (MASLD) [3,6]. Consequently, both direct and indirect mechanisms of COVID-19 have reinforced the need for long-term, non-invasive monitoring of liver health [7,8,9,10].

Multiparametric ultrasound (mpUS) has recently become an advanced radiologic modality capable of characterizing both mechanical and compositional liver properties. It integrates shear wave elastography (SWE) to assess stiffness and fibrosis, attenuation imaging (ATI) to quantify fat-related attenuation, and shear wave dispersion (SWD) to evaluate tissue viscosity potentially reflecting inflammation or compositional remodeling. The 2024 World Federation for Ultrasound in Medicine and Biology (WFUMB) guidelines define these techniques as complementary, quantitative tools for assessing liver disease [11,12,13]. SWE has achieved an established role in staging and prognostic evaluation of chronic liver disease, with liver stiffness measurement correlating with fibrosis stage and portal hypertension risk [11,14]. However, SWE readings may be influenced by inflammation, congestion, or elevated BMI [15,16]. Attenuation-based parameters, including ATI, allow quantification of ultrasound beam energy loss and are linked with hepatic fat fraction [17,18,19,20], while SWD adds value by detecting viscoelastic dispersion related to inflammation and/or hepatic fibrosis [14,21,22,23].

This study applies multiparametric ultrasound to evaluate long-term liver health in adults after COVID-19, focusing on SWE, ATI, and SWD parameters 3 years post-infection. Particular attention is given to SWE-defined fibrosis, ATI and SWD subgroup dynamics, and BMI categories. By combining advanced ultrasound metrics with clinical and metabolic data, this study aims to elucidate radiologic patterns of post-COVID liver changes and demonstrate the value of mpUS in long-term hepatic monitoring. A hypothesis was proposed that liver stiffness, attenuation, and dispersion parameters would change over a 3-year follow-up period in patients after the initial COVID-19 episode.

## 2. Materials and Methods

### 2.1. Study Design

This longitudinal prospective single-center study was approved by the appropriate ethics committee, and patient-informed consent was obtained according to the Declaration of Helsinki. The study was conducted between 2020 and 2023 and included 90 randomly selected adults hospitalized during their first COVID-19 episode (September–December 2020) and 39 clinically healthy control group who underwent thorough clinical and radiological baseline evaluation, and were then classified as research and control groups, respectively. Only 58 patients from research group, due to non-adherence, and all 39 patients from control group returned for a follow-up examination at an interval of 3 years.

However, during the 3-year follow-up period, 19 of the 39 patients in the control group were diagnosed with COVID-19, which was subsequently confirmed by serological testing, as evidenced by positive results for antibodies against the SARS-CoV-2 Nucleocapsid protein. Patients with previously known liver disease were excluded from the study. A physical examination of all patients was performed by a board-certified hepatologist (I.V.), and a structured questionnaire included information about the patients’ COVID-19 symptoms, including illness duration.

### 2.2. Patient Assessment

The baseline and follow-up assessment included laboratory workup with various biochemical indicators of hepatic injury. Baseline data were acquired within a 3–6-month period after the initial episode of COVID-19 and data were obtained from medical documentation and a questionnaire. It included social demographic data, comorbidities, characterization of COVID-19 severity, routine clinical tests, including liver tests (LT)—alanine aminotransferase (ALT) and/or aspartate aminotransferase (AST) and/or γ-glutamyltransferase (GGT) and/or lactate dehydrogenase (LDH),) and C-reactive protein (CRP), bilirubin fractions among others, as well as procalcitonin and D-dimer levers with additional screening for anti-SARS-CoV-2 IgM/IgG antibodies for all patients as proof of COVID-19 infection status.

### 2.3. Radiological Assessment

Radiological imaging consisted of a full abdominal ultrasound scan (Aplio i800 series US machine and the convex i8CX1 probe, Canon Medical Systems Corporation, Otawara, Japan), and the ultrasound scans were performed by a board-certified radiologist with more than 20 years of experience in abdominal imaging (M.R.) and a last-year resident radiologist (D.S.P.). To provide for reliable results, all patients were imaged using a standardized protocol for B-mode, color Doppler US, and multiparametric US elastography, with special attention paid to a guideline-adhering mid-breath hold supine intercostal hepatic approach. In addition to qualitative assessment of hepatic damage on B-mode ultrasound, both qualitative and quantitative parameters of the hepatic vasculature were evaluated on color Doppler US, including but not limited to portal vein dilation or altered flow parameters in the portal or hepatic veins, or hepatic artery.

The mpUS examination included 2-dimensional shear-wave elastography (2D-SWE) for quantifying increased liver stiffness, which was expressed in kPa following automatic conversion of shear wave propagation speed m/s^−1^ with Young’s modulus. No less than five measurements were performed within a homogeneous area of hepatic parenchyma and an interquartile range/median ratio (IQR/M) of 30% or less was used as an indicator for measurement reliability.

Increased liver stiffness was defined as SWE ≥ 7.1 kPa. In adjunct to 2D-SWE, shear-wave dispersion (SWD) was used to measure hepatic viscosity, calculated automatically in (m/s)/kHz during 2D-SWE scanning. Increased liver dispersion was defined as ≥12.0 (m/s)/kHz. Finally, attenuation imaging (ATI) was performed to quantitatively express the process of ultrasound wave attenuation by absorption, acoustic scattering and reflection, which increases proportionally to a higher fat fraction within hepatocytes.

Particularly to the ultrasound machine manufacturer, the ATI gen program instead of ATI pen was applied to avoid the potential of mismatched measurements. Increased liver attenuation was defined as ≥0.63 dB/cm/MHz. At follow-up, all patients underwent mpUS (repeated SWE, ATI, and SWD) on the same scanner system and repetitive biochemical testing. A summary of applied SWE, ATI and SWD values is depicted in Table 1 with the caveat that SWE analysis for the purposes of this study was using the ‘rule-of-four’ stratification recommended by WFUMB [12].

### 2.4. Statistical Analysis

Data analysis was performed using the SPSS 22.0 statistical package (IBM, New York, NY, USA). Normality of distribution was assessed by the Shapiro–Wilk test. For continuous variables, we performed Mann–Whitney test for group comparisons. Differences in categorical variables were detected using the Chi-squared method or two-tailed Fisher’s exact test for independent groups. Relationship between two continuous variables was analyzed using Pearson correlation test. For all analyses, a two-tailed *p*-value < 0.05 was considered statistically significant. All evaluations of the effect size included a 95% confidence interval (CI).

## 3. Results

### Main Results

**Figure 1:**
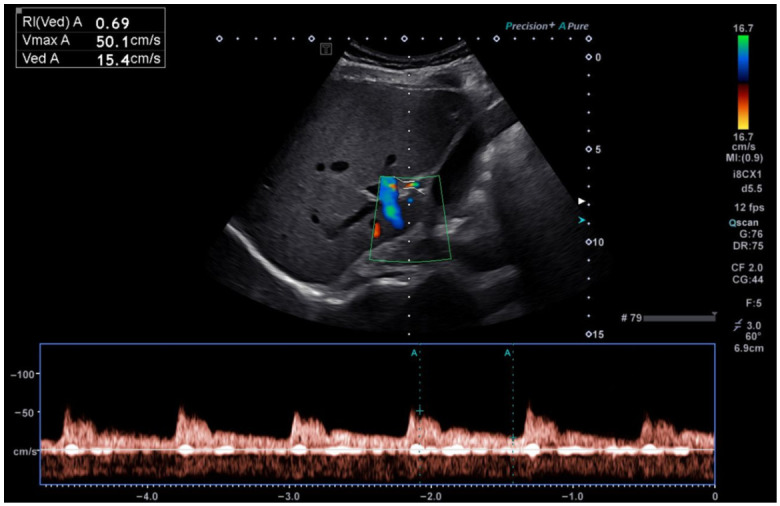
A color Doppler US evaluation for the same patient: in this case hepatic artery spectral analysis with normal flow parameters.

**Figure 2:**
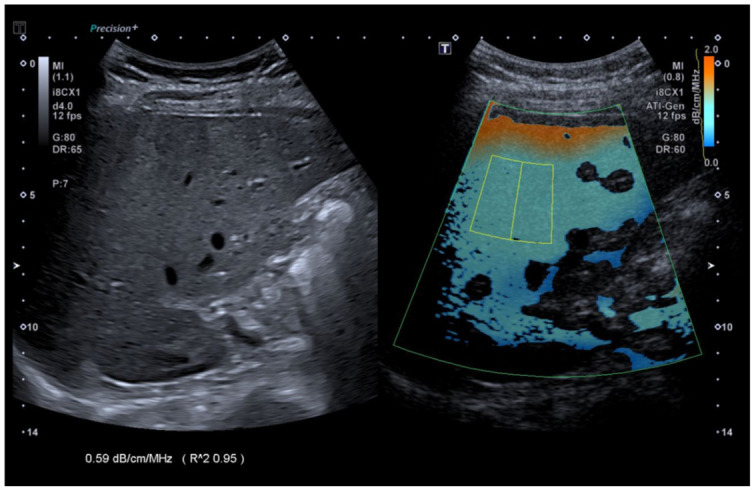
Attenuation imaging (ATI) with a gray-scale B-mode map (**left**) and an ATI value map (**right**), with the ROI (outlined in yellow) placed within the sample box below the near-field reverberation artifact zone (orange).

**Figure 3:**
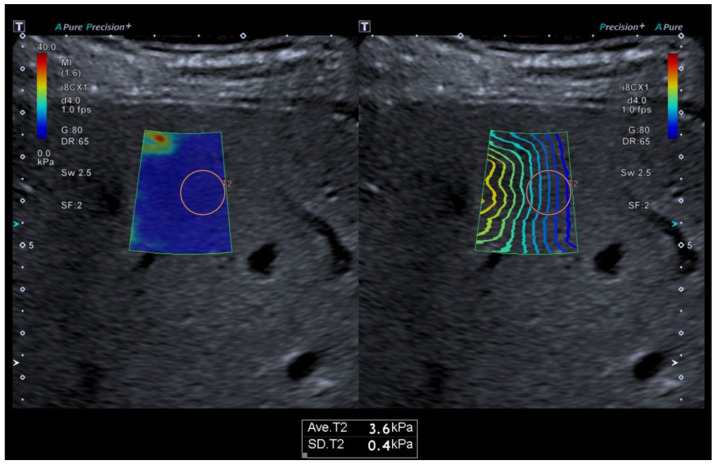
2D-SWE (shear-wave elastography) with sample-box maps overlaid on top of a B-mode image representing the SWE value (**left**) and shear wave propagation (**right**) maps in which wave propagation is displayed by parallel constantly spaced lines and an optimally placed region of interest (ROI) (pink circle) within a depth of approximately 4.0 cm.

**Figure 4:**
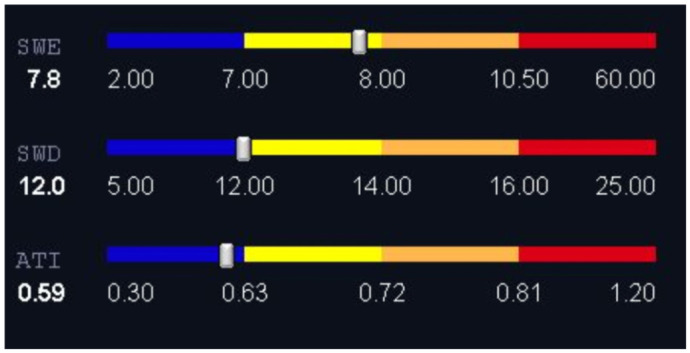
Case example of multiparametric ultrasound (mpUS) evaluation in patient with different results at initial and follow-up scan. MpUS in a female, 53 years of age (upon follow-up), with no history of previous liver disease. The initial mpUS scan revealed a slightly increased SWE value (median 7.8 kPa) and a borderline SWD value (12.0 (m/s)/kHz). Color coding of the horizontal bars defines severity grading of findings (blue—normal, yellow—light, orange—moderate, red—severe).

**Figure 5:**
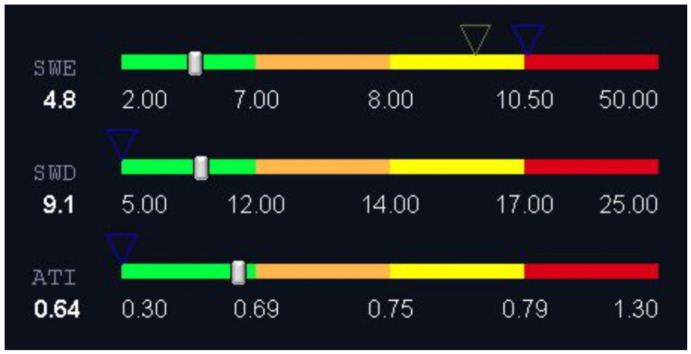
MpUS in the same female, 53 years of age (upon follow-up), with no history of previous liver disease. The follow-up mpUS scan 2 years and 7 months later revealed normal SWE and SWD values (respectively, 4.8 kPa and 9.1 (m/s)/kHz). Color coding of the horizontal bars defines severity grading of findings (green—normal, orange—light, yellow—moderate, red—severe), arrows indicate threshold values of SWE.

A total of 129 patients underwent baseline assessment. At the 3-year follow-up, a statistical cohort comprising 58 research group patients and 39 control group patients was analyzed, with a mean age of 46 ± 13 years (range 23–72) and 41.3–42.1 ± 10.1–10.9 years (range 26–62; 26–70), respectively. The main clinical, laboratory and imaging characteristics are summarized in Table 2 and Table 3. Representative imaging examples from the multiparametric ultrasound (mpUS) examinations are shown in Figure 1, Figure 2, Figure 3, Figure 4 and Figure 5.

The initial mpUS evaluation (baseline vs. 3-year follow-up) in the research group revealed a slight decrease in median SWE values from 5.04 ± 1.74 kPa (range 3.00–12.20) to 4.59 ± 0.81 kPa (range 2.60–6.20) at follow-up. Similarly, the median SWD decreased from 11.88 ± 1.73 m/s/kHz (range 8.80–18.70) to 10.83 ± 1.49 m/s/kHz (range 8.30–14.40). In contrast, median ATI values showed an increase over time, rising from 0.56 ± 0.09 dB/cm/MHz (range 0.39–0.85) to 0.60 ± 0.09 dB/cm/MHz (range 0.47–0.89). Despite these observable changes over time, the differences in SWE, SWD, and ATI values were not statistically significant (*p* > 0.05).

In the control group the initial median SWE was 4.55 ± 0.78 kPa (range 3.20–6.30), median dispersion slope was 10.80 ± 1.73 m/s/kHz (range 8.20–14.60), and median ATI was 0.57 ± 0.08 dB/cm/MHz (range 0.44–0.80).

At follow-up, control group patients were stratified according to COVID-19 status. In both the COVID-negative and -positive subgroup SWE slightly increased to 4.8 ± 0.88 kPa (range 3.30–6.50) and 4.7 ± 1.29 kPa (range 2.90–8.80), respectively (*p* > 0.05). Median SWD had a slight decrease to 10.15 ± 1.87 m/s/kHz (range 8.20–14.80) and 10.6 ± 1.82 m/s/kHz (range 6.30–14.30), respectively (*p* > 0.05). However, in both subgroups ATI increased to 0.62 ± 0.09 dB/cm/MHz (range 0.50–0.82) and 0.65 ± 0.07 dB/cm/MHz (range 0.52–0.77), with statistical significance (*p* < 0.05), respectively. Furthermore, in both COVID-negative and -positive subgroups, there was a statistically significant (*p* < 0.001) increase in the BMI from a baseline result of 25.0 ± 3.5 (range 18.4–32.0) to 28.7 ± 6.2 (19.80–49.70) and 30.5 ± 5.1 (22.30–39.20), respectively.

Higher hepatic stiffness values were found to have a statistically significant positive correlation with higher levels of biomarkers of liver injury, including GGT (rs = 0.37, *p* = 0.005), ALT (rs = 0.31, *p* = 0.02) as well as BMI (rs = 0.49, *p* < 0.001). A mild positive correlation was revealed between SWE and SWD values, with higher median stiffness values corresponding to higher values of dispersion (OR = 1.407; *p* = 0.001; 95% CI 1.011–1.960). Increased ATI values correlated to increased BMI (*p* < 0.001).

## 4. Discussion

In our study, all patients exhibited a long-term increase in ultrasound attenuation values, indicative of the development of increased hepatic fat accumulation, irrespective of COVID-19 status, even in patients without prior liver disease. In the acute phase, more than half of patients with non-severe and over 80% of patients with severe or critical COVID-19 exhibited liver test (LT) abnormalities, consistent with previous studies reporting mild to moderate hepatic injury during SARS-CoV-2 infection [1,5]. The exclusion of viral, autoimmune, and metabolic liver diseases suggests that most LT abnormalities observed during hospitalization were acquired during the acute infection, reflecting direct viral cytopathic effects, systemic inflammation, and oxidative hepatocellular stress [1,2,4].

It has been reported that COVID-19-related high CRP levels correlate strongly with elevated SWD values, suggestive of prolonged inflammation up to 3–6 months [24]. Over 3 years of follow-up, biochemical indicators of hepatic injury largely normalized; however, quantitative imaging revealed that a subset of patients developed radiologically measurable alterations in liver tissue. MpUS proved to be valuable in quantifying these changes, predominantly in those who had experienced a severe or critical disease course, potentially reflecting long-term hepatic injury described in recent longitudinal cohorts [8,9,10]. Concurrently, attenuation imaging (ATI) identified steatosis in up to 61% of severe cases, suggesting that hepatic fat accumulation may represent a long-term metabolic sequela of COVID-19. These findings are consistent with emerging reports of post-COVID hepatic steatosis and necroinflammation assessed by non-invasive imaging modalities [5,8,9,21]. The observed improvement in biochemical parameters with persistent mpUS abnormalities highlights the importance of radiologic tissue characterization beyond conventional liver tests. Elastography-based stiffness and dispersion parameters can detect subclinical fibrotic and inflammatory alterations [25,26,27,28] even after normalization of transaminases [11,14,17]. This discordance emphasizes the role of mpUS as a follow-up imaging tool, complementing serum-based fibrosis indices such as FIB-4, especially in metabolic or post-viral contexts. Our data align with recent WFUMB recommendations, which support the integration of SWE, SWD, and ATI into comprehensive liver assessments [11,12,13]. The higher ATI values in patients with a history of severe COVID-19 indicate both residual stiffness and parenchymal attenuation consistent with fatty infiltration and extracellular matrix accumulation, and although other factors, such as hepatic fibrosis or hepatic venous congestion, could alter the measured values, patients were screened for hepatic fibrosis clinically, and hepatic and systemic Doppler-US did not reveal signs of hepatic venous congestion in any of the patients. Notably, SWD showed a trend toward higher values in severe disease, suggesting persistent microviscosity alterations, possibly a marker of low-grade inflammation or post-injury remodeling [17,21,22,23].

Importantly, our cohort analysis also revealed that metabolic and cardiovascular comorbidities contribute to persistent liver changes. Nearly half of the cohort was overweight or obese, and patients with cardiovascular disease demonstrated higher SWE values at follow-up. Regression analysis confirmed cardiovascular disease and age as independent predictors of increased liver stiffness, consistent with the established link between metabolic dysfunction, vascular alterations, and hepatic fibrogenesis [3,5,15,16].

From a radiologic perspective, mpUS offers several advantages. It provides real-time, quantitative assessment of stiffness, attenuation, and dispersion—each reflecting distinct tissue components (fibrosis, fat, and viscosity)—and has been shown to be a useful method for follow-up in other studies [25,29,30]. In contrast to serum markers, mpUS can spatially localize and characterize parenchymal heterogeneity. This makes it particularly suitable for follow-up evaluations, where subtle mechanical or compositional alterations may precede biochemical changes. The complementary use of SWE, ATI, and SWD thus enhances diagnostic precision and supports risk stratification in long-term COVID-related liver monitoring [11,17,21,22,23].

The present study demonstrates that initially COVID-19-affected patients’ ATI values remained stable over time, corresponding with unchanged BMI values, and there was no evidence of hepatic steatosis in the research group. Minor reduction in dispersion values was observed at follow-up imaging, suggestive of resolving inflammatory components and no signs of fibrosis development median SWE values remained within normal range. In contrast, the control group exhibited a significant increase in median ATI values over time, especially in COVID-19-later-affected control group individuals corresponding to development of mild steatosis, whereas non-affected individuals showed subthreshold values. This increase is more plausibly explained by changes in BMI increase and metabolic disorder rather than by direct virus-induced hepatic damage. The stability of dispersion values and SWE measurement remained within the normal range, confirming absence of significant inflammatory component or liver fibrosis.

Nevertheless, this study has limitations. The sample size was moderate, constrained by pandemic-era logistics and long-term follow-up losses. Additionally, the absence of baseline imaging before COVID-19 precludes absolute differentiation between de novo and pre-existing steatosis. Still, the exclusion of patients with known chronic liver diseases and the longitudinal prospective design strengthen the causal inference between COVID-19 and subsequent radiologic findings [7,8].

## 5. Conclusions

In conclusion, our findings provide no evidence of persistent liver damage attributable to COVID-19 and observed variations in mpUS hepatic parameters in long-term are more likely related to lifestyle rather than direct lasting viral impact.

## Figures and Tables

**Table 1:** Liver stiffness by shear wave elastography (SWE), dispersion (SWD) and attenuation tissue imaging (ATI) groups with cut-off values provided by Canon Medical.

	Normal	Mild	Moderate	Severe
SWE, kPa *	<7.00	7.00–8.00	8.00–10.50	>10.50
SWD, (m/s)/kHz	<12.00	12.00–14.00	14.00–17.00	>17.00
ATI, dB/cm/Mhz	<0.69	0.69–0.75	0.75–0.79	>0.79

* data for the study stratified using ‘rule-of-four’ as recommended by WFUMB.

**Table 2:** Research group parameters, main clinical, laboratory and imaging data including initial and follow-up examination.

Research Group	Initial Examination	Follow-Up
Patients	*n* = 90	*n* = 58
Age, years (range)	43.3 ± 13 (23–69)	46.0 ± 13 (26–72)
Gender; female/male, *n* (%)	48/42 (53%/47%)	28/30 (48%/52%)
Months since COVID-19, *n*	6.4 ± 1.9 (3.0–9.0)	36.4 ± 1.9 (34.0–39.0)
Duration of COVID-19, months	1.3 ± 0.6 (0.2–3.0)	1.3 ± 0.6 (0.2–3.0)
Hospitalization rate, *n* (%)	34 (60%)	NA
Disease severity, *n* (%)		
Mild	13 (43%)	NA
Moderate	10 (33%)	NA
Severe	7 (23%)	NA
BMI, kg/m^2^ (range)	27.2 ± 4.8 (19.7–40.3)	27.2 ± 5.4 (18.3–41.8)
**Biochemical profile**		
ALT, U/L [0–55 U/L] (range)	22.6 (14.0–186.0)	21.8 (11.0–129.0)
AST, U/L [5–35 U/L] (range)	32.3 (10.0–122.0)	28.9 (13.0–60.0)
GGT, U/L [12–64 U/L] (range)	31.0 (6.0–118.0)	31.0 (3.0–126.0)
LDH, U/L [125–220 U/L] (range)	173.9 (72.0–344.0)	176.4 (103.0–251.0)
CRP, mg/L [0–5 mg/L] (range) *	20.5 (1.0–57.0)	2.1 (0.1–20.1)
**Imaging findings**		
Median SWE value, kPa (range) **	5.04 ± 1.74 (3.00–12.20)	4.59 ± 0.81 (2.60–6.20) (*p* > 0.05)
Median dispersion value, (m/s)/kHz (range) **	11.88 ± 1.73 (8.80–18.70)	10.83 ± 1.49 (8.30–14.40) (*p* > 0.05)
Median ATI value, dB/cm/MHz (range) **	0.56 ± 0.09 (0.39–0.85)	0.60 ± 0.09 (0.47–0.89) (*p* > 0.05)

** data presented as mean ± SD. SD, standard deviation; range (from minimum value to maximum value); BMI, body mass index; AST, aspartate transaminase; ALT, alanine transaminase; GGT, gamma-glutamyl transferase; LDH, lactate dehydrogenase; CRP, C-reactive protein. SWE, shear-wave elastography; ATI, attenuation imaging; NA—not applicable; * results with a *p* value of assumed significance < 0.05.

**Table 3:** Control group parameters, main clinical, laboratory and imaging data including initial and follow-up examination.

Control Group	Initial Examination	Follow-Up *** (COVID-Negative)	Follow-Up *** (COVID-Positive)
Patients	*n* = 39	*n* = 20	*n* = 19
Age, years (range)	39.5 ± 12.9 (23–67)	41.3 ± 10.1 (26–62)	42.1 ± 10.9 (26–70)
Gender; female/male, *n* (%)	33/6 (84%/16%)	17/3 (85%/15%)	16/3 (84%/16%)
BMI, kg/m^2^ (range)	25.0 ± 3.5 (18.4–32)	28.7 ± 6.2 (19.80–49.70)	30.5 ± 5.1 (22.30–39.20)
**Biochemical profile**			
ALT, U/L [0–55 U/L] (range)	28.4 (16.0–84.0)	27.65 (13.0–89.0)	31.17 (15.2–108.0)
AST, U/L [5–35 U/L] (range)	21.4 (15.0–32.0)	21.08 (13.0–31.0)	26.51 (14.4–42.2)
GGT, U/L [12–64 U/L] (range)	31.0 (13.9–101.0)	28.13 (13.0–95.0)	35.77 (19.0–135.0)
LDH, U/L [125–220 U/L] (range)	179.5 (130.0–236.0)	175.43(108.0–219.0)	181.59 (121.0–225.0)
CRP, mg/L [0–5 mg/L] (range)	3.0 (1.0–5.4)	2.5 (0.1–6.2)	3.3 (1.2–5.7)
**Imaging findings**			
Median SWE value, kPa (range) **	4.55 ± 0.78 (3.20–6.30)	4.8 ± 0.88 (3.30–6.50) (*p* > 0.05)	4.7 ± 1.29 (2.90–8.80) (*p* > 0.05)
Median dispersion value, (m/s)/kHz (range) **	10.80 ± 1.73 (8.20–14.60)	10.15 ± 1.87 (8.20–14.80) (*p* > 0.05)	10.6 ± 1.82 (6.30–14.30) (*p* > 0.05)
Median ATI value, dB/cm/MHz (range) **	0.57 ± 0.08 (0.44–0.80)	0.62 ± 0.09 (0.50–0.82) * (*p* < 0.05)	0.65 ± 0.07 (0.52–0.77) * (*p* < 0.05)

** data presented as mean ± SD. SD, standard deviation; range (from minimum value to maximum value); BMI, body mass index; AST, aspartate transaminase; ALT, alanine transaminase; GGT, gamma-glutamyl transferase; LDH, lactate dehydrogenase; CRP, C-reactive protein. SWE, shear-wave elastography; ATI, attenuation imaging. *** as defined by either a positive (+) or negative (−) COVID-19 N protein test. * results with a *p* value of assumed significance < 0.05.

## Data Availability

The datasets analyzed during the current study are available from the corresponding author on reasonable request.

## Abbreviations

The following abbreviations are used in this manuscript:

2D-SWE	2-dimensional shear-wave elastography
SWD	shear-wave dispersion
ATI	attenuation imaging
COVID-19	coronavirus disease 2019
mpUS	multiparametric ultrasound
SD	standard deviation
SE	standard error
BMI	body mass index
MASLD	metabolic dysfunction-associated steatotic liver disease
WFUMB	World Federation for Ultrasound in Medicine and Biology
AlT	alanine transaminase
AsT	aspartame transaminase
LDH	lactate dehydrogenase
GGT	gamma-glutamyl transferase
CRP	C-reactive protein
IQR/M	interquartile range/median ratio
LT	liver test

## References

[1] Marjot T., Webb G.J., Barritt A.S., Moon A.M., Stamataki Z., Wong V.W., Barnes E. (2021). COVID-19 and liver disease: Mechanistic and clinical perspectives. Nat. Rev. Gastroenterol. Hepatol..

[2] McConnell M.J., Kondo R., Kawaguchi N., Iwakiri Y. (2022). Covid-19 and Liver Injury: Role of Inflammatory Endotheliopathy, Platelet Dysfunction, and Thrombosis. Hepatol. Commun..

[3] Eslam M., Newsome P.N., Sarin S.K., Anstee Q.M., Targher G., Romero-Gomez M., Zelber-Sagi S., Wong V.W.-S., Dufour J.-F., Schattenberg J.M. (2020). A new definition for metabolic dysfunction-associated fatty liver disease: An international expert consensus statement. J. Hepatol..

[4] Roshanshad R., Roshanshad A., Fereidooni R., Hosseini-Bensenjan M. (2023). COVID-19 and liver injury: Pathophysiology, risk factors, outcome and management in special populations. World J. Hepatol..

[5] Li X., Fan C., Tang J., Zhang N. (2023). Meta-analysis of liver injury in patients with COVID-19. Medicine.

[6] Younossi Z.M., Marchesini G., Pinto-Cortez H., Petta S. (2019). Epidemiology of Nonalcoholic Fatty Liver Disease and Nonalcoholic Steatohepatitis: Implications for Liver Transplantation. Transplantation.

[7] Brandi N., Spinelli D., Granito A., Tovoli F., Piscaglia F., Golfieri R., Renzulli M. (2023). COVID-19: Has the Liver Been Spared?. Int. J. Mol. Sci..

[8] Zhang C., Shi L., Wang F.S. (2020). Liver injury in COVID-19: Management and challenges. Lancet Gastroenterol. Hepatol..

[9] Taylor-Robinson S.D., Morgan M.Y. (2023). COVID-19 and the Liver: A Complex and Evolving Picture. Hepat Med..

[10] Aby E.S., Moafa G., Latt N., Sultan M.T., Cacioppo P.A., Kumar S., Chung R.T., Bloom P.P., Gustafson J., Daidone M. (2023). Long-term clinical outcomes of patients with COVID-19 and chronic liver disease: US multicenter COLD study. Hepatol. Commun..

[11] Ferraioli G., Wong V.W., Castera L., Berzigotti A., Sporea I., Dietrich C.F., Choi B.I., Wilson S.R., Kudo M., Barr R.G. (2018). Liver Ultrasound Elastography: An Update to the World Federation for Ultrasound in Medicine and Biology Guidelines and Recommendations. Ultrasound Med. Biol..

[12] Ferraioli G., Barr R.G., Berzigotti A., Sporea I., Wong V.W.-S., Reiberger T., Karlas T., Thiele M., Cardoso A.C., Ayonrinde O.T. (2024). WFUMB Guideline/Guidance on Liver Multiparametric Ultrasound: Part 1. Update to 2018 Guidelines on Liver Ultrasound Elastography. Ultrasound Med. Biol..

[13] Ferraioli G., Barr R.G., Berzigotti A., Sporea I., Wong V.W.-S., Reiberger T., Karlas T., Thiele M., Cardoso A.C., Ayonrinde O.T. (2024). WFUMB Guidelines/Guidance on Liver Multiparametric Ultrasound. Part 2: Guidance on Liver Fat Quantification. Ultrasound Med. Biol..

[14] Thiele M., Madsen B.S., Procopet B., Hansen J.F., Møller L.M.S., Detlefsen S., Berzigotti A., Krag A. (2017). Reliability Criteria for Liver Stiffness Measurements with Real-Time 2D Shear Wave Elastography in Different Clinical Scenarios of Chronic Liver Disease. Reliabilitätskriterien für die Messung der Lebersteifigkeit mittels Echtzeit-2D-Shearwave-Elastografie bei verschiedenen klinischen Szenarien der chronischen Lebererkrankung. Ultraschall Med..

[15] Zeng J., Huang Z.P., Zheng J., Wu T., Zheng R.Q. (2017). Non-invasive assessment of liver fibrosis using two-dimensional shear wave elastography in patients with autoimmune liver diseases. World J. Gastroenterol..

[16] Mendoza Y.P., Rodrigues S.G., Delgado M.G., Murgia G., Lange N.F., Schropp J., Montani M., Dufour J.-F., Berzigotti A. (2022). Inflammatory activity affects the accuracy of liver stiffness measurement by transient elastography but not by two-dimensional shear wave elastography in non-alcoholic fatty liver disease. Liver Int..

[17] Chattopadhyay T., Chan H.-J., Le D.C., Wang C.-Y., Tai D.-I., Zhou Z., Tsui P.-H. (2025). Multiparametric Quantitative Ultrasound as a Potential Imaging Biomarker for Noninvasive Detection of Nonalcoholic Steatohepatitis: A Clinical Feasibility Study. Diagnostics.

[18] Kaliaev A., Chavez W., Soto J., Huda F., Xie H., Nguyen M., Shamdasani V., Anderson S. (2022). Quantitative Ultrasound Assessment of Hepatic Steatosis. J. Clin. Exp. Hepatol..

[19] Nishimura T., Tada T., Akita T., Kondo R., Suzuki Y., Imajo K., Kokubu S., Abe T., Kuroda H., Hirooka M. (2025). Diagnostic performance of attenuation imaging versus controlled attenuation parameter for hepatic steatosis with MRI-based proton density fat fraction as the reference standard: A prospective multicenter study. J. Gastroenterol..

[20] Hänni O., Ruby L., Paverd C., Frauenfelder T., Rominger M.B., Martin A. (2024). Comparison of Ultrasound Attenuation Imaging Using a Linear versus a Conventional Convex Probe: A Volunteer Study. Diagnostics.

[21] Seneviratne N., Fang C., Sidhu P.S. (2023). Ultrasound-based hepatic fat quantification: Current status and future directions. Clin. Radiol..

[22] Yin M. (2024). Shear-Wave Dispersion for Detecting Hepatic Inflammation in Metabolic Dysfunction-associated Steatotic Liver Disease. Radiology.

[23] Grgurevic I., Tjesic Drinkovic I., Pinzani M. (2019). Multiparametric ultrasound in liver diseases: An overview for the practising clinician. Postgrad. Med. J..

[24] Radzina M., Putrins D.S., Micena A., Vanaga I., Kolesova O., Platkajis A., Viksna L. (2022). Post-COVID-19 Liver Injury: Comprehensive Imaging With Multiparametric Ultrasound. J. Ultrasound Med..

[25] Choaib A., Issa E., El Choueiry F., Eldin J.N., Shbaklo K., Alhajj M., Sawaya R.T., Assi G., Nader M., Chatila R. (2023). SARS-CoV-2-mediated liver injury: Pathophysiology and mechanisms of disease. Inflamm. Res..

[26] Heidari F., Pierce T., Sertic M., Hegde S., Hunt D., Ozturk A., Samir A.E. (2024). Lasting liver injury following COVID-19 infection characterized by ultrasound shear wave elastography. WFUMB Ultrasound Open.

[27] Rodriguez-Espada A., Salgado-de la Mora M., Rodriguez-Paniagua B.M., Limon-de la Rosa N., Martinez-Gutierrez M.I., Pastrana-Brandes S., Navarro-Alvarez N. (2024). Histopathological impact of SARS-CoV-2 on the liver: Cellular damage and long-term complications. World J. Gastroenterol..

[28] Moreira J.L.S., Barbosa S.M.B., Gonçalves Junior J. (2021). Pathophysiology and molecular mechanisms of liver injury in severe forms of COVID-19: An integrative review. Clin. Res. Hepatol. Gastroenterol..

[29] Effenberger M., Grander C., Fritsche G., Bellmann-Weiler R., Hartig F., Wildner S., Seiwald S., Adolph T.E., Zoller H., Weiss G. (2020). Liver stiffness by transient elastography accompanies illness severity in COVID-19. BMJ Open Gastroenterol..

[30] Hu D., Fu H., Meng H., Zhao J. (2023). Evaluation of liver stiffness using ultrasonic shear wave elastography in patients with COVID-19 induced pneumonia. Can. J. Physiol. Pharmacol..

